# Investigation of Natural Resistance to Fostemsavir and Lenacapavir in Naïve Primary Infections by Ultra-Deep Sequencing of near Full-Length HIV-1 Genomes

**DOI:** 10.3390/v17050636

**Published:** 2025-04-28

**Authors:** Elisabetta Lazzari, Gabriella Rozera, Roberta Gagliardini, Valentina Mazzotta, Lavinia Fabeni, Federica Forbici, Giulia Berno, Cristian Cosentino, Enrico Girardi, Andrea Antinori, Fabrizio Maggi, Isabella Abbate

**Affiliations:** 1 Laboratory of Virology, National Institute for Infectious Diseases Lazzaro Spallanzani—IRCCS, 00149 Rome, Italy; elisabetta.lazzari@inmi.it (E.L.); lavinia.fabeni@inmi.it (L.F.); federica.forbici@inmi.it (F.F.); giulia.berno@inmi.it (G.B.); fabrizio.maggi@inmi.it (F.M.); isabella.abbate@inmi.it (I.A.); 2 Clinical Department, National Institute for Infectious Diseases Lazzaro Spallanzani—IRCCS, 00149 Rome, Italy; roberta.gagliardini@inmi.it (R.G.); valentina.mazzotta@inmi.it (V.M.); andrea.antinori@inmi.it (A.A.); 3 QIAGEN Aarhus, 8000 Aarhus, Denmark; cristian.cosentino@qiagen.com; 4 Scientific Direction, National Institute for Infectious Diseases Lazzaro Spallanzani—IRCCS, 00149 Rome, Italy; enrico.girardi@inmi.it

**Keywords:** HIV, drug resistance, quasispecies, antiretroviral therapy

## Abstract

Next-generation sequencing (NGS) of near full-length HIV genomes was performed to investigate natural resistance to Fostemsavir (FTR) and Lenacapavir (LEN) at the quasispecies level in nine naïve primary HIV infections harboring different HIV subtypes and recombinant forms. Reconstructed genomes provided a median (IQR) coverage for *gag* and *env* of 1710 (750–6063) and 1768 (871–5270), respectively. In the gp120 encoding region, the M426R variant was found with a frequency of 100% in two HIV subtypes B: one of these also showed the A204T variant at 100%. In the more conserved capsid coding region no mutations possibly related to LEN natural resistance were observed.

## 1. Introduction

Heavily treatment-experienced (HTE) individuals with human immunodeficiency virus (HIV)-1 have limited therapeutic options due to multidrug resistance, drug intolerance or toxicity, and comorbidities. To face the emergence of drug-resistant strains during combined antiretroviral treatment (cART), new HIV antiretroviral drugs are continuously under study and evaluation [1]. Recently, two new drugs have been introduced for HTE that have no potential cross-resistance with previously administered regimes. Fostemsavir (FTR), commercial name Rukobia, received US approval in 2020 (in Europe in 2021) (FDA https://www.fda.gov, EMA http://www.ema.europa.eu, last accessed 27 August 2024), while Lenacapavir (LEN), commercial name Sunlenca, was approved in the EU for the treatment of HIV-positive adults with multidrug resistance in 2022 in both Europe and the USA [2,3,4]. FTR is a novel pre-attachment inhibitor that interferes with the interaction between HIV glycoprotein 120 (gp120) and the CD4 receptor, impeding viral entry in the host T cell. More specifically, it attaches close to the CD4 binding loop and β20-β21 hairpin/V3 region, stabilizing env in a ‘closed’ state [3]. LEN is the first drug targeting viral capsid (CA) protein 24 (p24) and is a hindrance to multiple stages of the viral replication cycle, such as nuclear uptake of the pre-integration complex and virion morphogenesis [4]. This novel drug competes for the CA binding site with several host-cell nuclear import cofactors, preventing the nuclear import of viral cDNA and disrupting the CA flexibility and functional disassembly required for the formation of an infectious virus [5]. Currently, data regarding natural resistance—i.e., the presence of resistance-associated mutations in patients who have never received antiretroviral therapy, potentially due to transmitted resistance or naturally occurring polymorphisms—to FTR and LEN concerns the analysis of population sequences [6,7,8]. Natural resistance to FTR was linked to mutations identified among several HIV-1 subtypes, including major groups A, B, C, D, F, G, and H, and circulating recombinant forms (CRFs) such as CRF01_AE, CRF02_AG, and CRF03_AB [6]. LEN mutations associated with natural drug resistance were found in HIV-1 subtype C and CRF01_AE [8]. Nothing to our knowledge has been reported about the presence of minority variants linked to a probable natural drug resistance at the viral quasispecies level among people living with HIV (PLWH) yet.

## 2. Materials and Methods

### 2.1. Study Population and Virological Assessment

To investigate possible natural resistance to FTR and LEN being present as minority variants in the viral quasispecies, nine primary HIV infections (PHI) consecutively enrolled in the observational cohort of primary infection (SIREA cohort) of the National Institute for Infectious Diseases (INMI) of Rome, Italy, were studied. Serodiagnosis of PHI subjects was posed based on either the combination of an HIV Ab/Ag Combo positive (Abbott Alinity HIV Ag/Ab Combo assay, Abbott Park, IL, USA), and an Immuno Blot (Geenius, BioRad, Bio-Rad Laboratories S.r.l. Milan, Italy) negative assay with a positive HIV-1 RNA test or an HIV Ab/Ag Combo positive and Immuno Blot indeterminate assay, according to WHO criteria for Western blot confirmatory assay, i.e., two env reactive bands. Plasma HIV-1 RNA was measured by Aptima™ HIV-1 Quant Dx Assay (Hologic, Inc., San Diego, CA, USA). HIV-1 subtyping was established through molecular phylogenetic analysis of pol sequences performed for drug resistance genotyping. The subtype assignments were validated using the HIV Resistance Database (https://hivdb.stanford.edu/) and confirmed via the COMET subtyping tool (https://comet.lih.lu/ last accessed on 17 April 2025), as previously described [9]. Prediction of HIV-coreceptor usage by the analysis of the V3 region was performed using Geno2Pheno software (https://www.geno2pheno.org/, last accessed 27 August 2024). The SIREA study was approved by the INMI Ethical Committee on 18 February 2014, while control samples were residuals of diagnostic evaluations (Ethical Committee, CET Lazio area 4 approval number 61/2023, 4 December 2023). All subjects were requested to sign an informed consent.

### 2.2. Near-Full-Length PCR and Bioinformatic Analysis

Near-full-length genomes were obtained by fragmentation and NGS of two large HIV genome amplicons. Briefly, after nucleic extraction, RNA was submitted to retrotranscription using oligo-dT and pan-subtyping oligonucleotides, as described in Grossman et al. [10]. The obtained cDNA was then amplified through nested and semi-nested PCR, using pan-subtype primers [10], providing two large fragments of almost 5000 bp and 3000 bp in length that underwent NGS using Novaseq platform of Illumina technology, (San Diego, CA, USA) and a paired-end 150 bp × 2 approach. For the reconstruction of all near-full-length genomes, an in-house bioinformatic pipeline was created using QIAGEN CLC Genomics Workbench 24.0 software (https://digitalinsights.qiagen.com/ last accessed on 17 April 2025; extended with the Biomedical Genomics Analysis 24.01 module). The pipeline first trimmed reads by quality and adapter sequences, and then the cleaned reads were mapped to the HIV subtype B HXB2 reference sequence (GenBank K03455). Reads with a coverage below 30 were not considered for the consensus sequence construction. Read depth statistics were computed for each CDS region of HXB2. Variants were called through the Low Frequency Variant Detection tool, which performed a statistical test to determine if the nucleotides observed at a given site could be due to sequencing errors or real alleles and annotated with overlap information from CDS annotations of HXB2. Detection threshold was set with a minimum coverage of 10 and a minimum frequency of 3%.

Minority amino acid variants were identified by aligning the reference genome nucleotide sequence with its coding sequence (CDS) annotation and the reconstructed HIV genome from patient samples. When present, the frequency of each variant was calculated as the ratio of the nucleotide variant count to the total read coverage at the corresponding genomic position.

Polymorphisms, i.e., naturally occurring sequence variations not necessarily associated with drug resistance- and minor mutations, i.e., low-frequency variants potentially conferring resistance, for possible resistance to the FTR and LEN were finally evaluated.

## 3. Results

**Figure 1:**
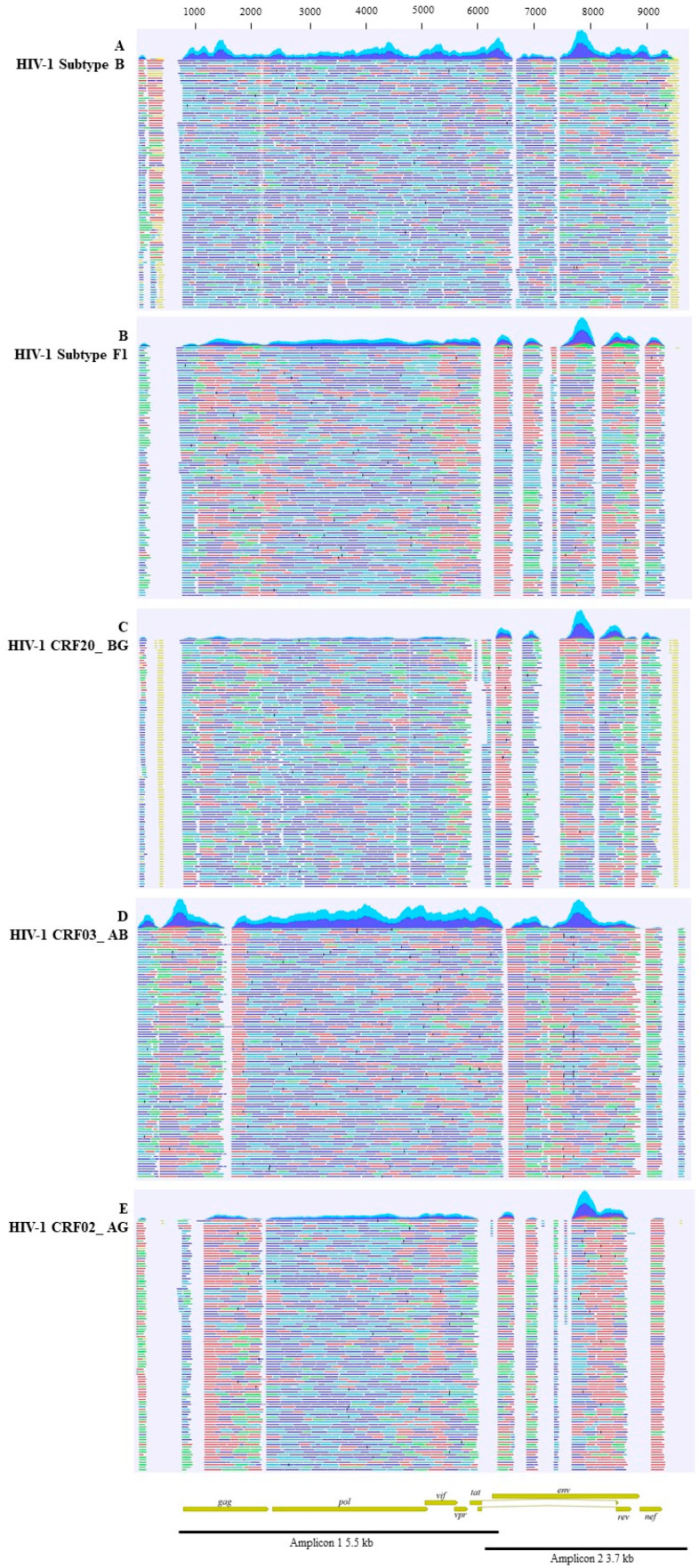
Near full-length genomes with the first 100 contigs reconstructed and aligned to the reference genome for each considered virus. Panels (**A**–**E**) display a representative HIV-1 subtype B (sample HIV-1_B_62), HIV-1 subtype F1, HIV-1 CRF20_BG, HIV-1 CRF03_AB, and HIV-1 CRF02_AG, respectively. On top of each panel, sequencing coverage peaks are shown. Below the coverage, different colors are used to describe the reads paired to their own reference HIV sequence: forward correctly paired reads are shown in dark blue; reverse correctly paired reads are shown in light blue; forward unpaired reads are displayed in green; and reverse unpaired reads are displayed in red. Additionally, non-specific matches are represented in yellow. Below the panels, HIV-1 genome annotation is reported, with the size of the two PCR amplicons used for NGS.

HIV subtyping provided as a result five HIV-1 subtype B and one of HIV-1 subtype F1, CRF03_AB, CRF20_BG, and CRF02_AG, respectively. All displayed a median (IQR) plasma HIV-1 RNA 6.97 (5.87–7.49) Log copies/ml. Starting from NGS data and using the constructed ad hoc bioinformatic pipeline with the QIAGEN CLC Genomics Workbench 24.0 software, nine near-full-length genomes, approximately from nucleotide 1 to nucleotide 9719, were obtained, with a median (IQR) read depth of 3524 (930–6520). In Figure 1, the first 100 reconstructed contigs are shown for each subtype type studied: one representative for subtype B (panel A) and the others for subtype F1 (panel B), CRF20_BG (panel C), CRF03_AB (panel D), and CRF02_AG (panel E). For each panel, coverage peaks, associated with regions with higher sequencing depth, are displayed on top. The *gag* and *env* regions displayed a median (IQR) read depth of 1710 (750–6063) and 1768 (871–5270), respectively. As expected, reconstructed non-B HIV genomes presented several gaps due to the unique reference sequence used for all (HXB2) to subsequently investigate amino acid variants possibly associated with drug resistance. However, the entire *gag* and *env* regions were always entirely reconstructed. The investigation for the possible presence of natural resistance was manually done by comparing the obtained amino acid sequences at each position to known substitutions that are currently recognized to confer genotypic resistance to FTR and LEN. For a complete list of these variants, see Table 1. Both mutations found in patients who develop drug resistance in clinical studies, such as BRIGHTE [11] and CAPELLA [12], and other mutations described to rise during in vitro experiments conducted in the presence of the drug were considered. The BAM files of the aligned reads have been submitted to NCBI Sequence Reads Archive (SRA), accession number PRJNA1144728. The results indicated that in the gp120 encoding region, the M426R variant was found with a frequency of 100% in two HIV subtypes B: one of these also showed the A204T variant at 100%. The analysis of the V3 region indicated that one PHI with the M426R variant displayed a prevalent R5 tropism (False Detection Rate, FDR 59.8%), while the other with both M246R and A204T variants showed a prevalent X4 viral tropism (FDR 1.3%). The analysis of the gag region at the quasispecies level did not identify, in the present study population, either mutations known to confer clinical resistance or any polymorphism among the variants reported in Table 1.

## 4. Discussion

In the present study NGS of near full-length HIV genomes was performed to investigate natural resistance to FTR and LEN at the quasispecies level in naïve HIV infections. PHI investigation was chosen since it provides a more realistic snapshot of the circulating variants, being the analysis of the viral genome very close to the time of transmission.

The results indicated, in the quasispecies of the analyzed PHI, a generally higher degree of heterogeneity in *env* with respect to the *gag* region.

Regarding amino acid variants possibly associated with FTR drug resistance, in two HIV subtype B, a polymorphism at position 426 of the protein sequence was detected. While the substitution associated with a very important clinical drug resistance is M426L [7,13], the substitution M426R at a frequency of 100% in both samples was observed. This polymorphism was already found to be recurrent in HIV B-subtypes [6,7]. In one of these two B subtypes, at a frequency of 100%, another substitution in position 204, namely the A204T, was also found. At this position, another substitution, A204D, is known to be associated with in vitro resistance/decreased susceptibility to FTR [7]. Lepore and colleagues showed that the presence of both of these two substitutions is linked to both HIV subtype B and viral tropism. More specifically, tropism M426R is associated with an R5 tropism, while A204T is linked to an X4 viral [14]. According to these findings, the analysis of the V3 region indicated that one PHI with the M426R variant displayed a prevalent R5 tropism, while the other with both M246R and A204T variants showed a prevalent X4 viral tropism. In the more conserved capsid coding region no mutations possibly related to LEN natural resistance were observed.

In conclusion, although an ultra-sensitive method was used to detect natural resistance to FTR and LEN, no possible natural resistance was appreciated. However, the present data confirm that at the quasispecies level, the env region displays a higher genetic heterogeneity with respect to the *gag* region. This is also true if the FTR binding site of gp120 is adjacent to the region that has to bind the conserved structure of the CD4 receptor. On the other hand, the high level of amino acid conservation in the *gag* region coding for CA can be attributed to the key role of this protein in the multistep processes of the HIV replication cycle, making any amino acid substitutions disadvantageous for viral propagation. The presence of minority variants linked to natural drug resistance in the viral quasispecies may be considered a risk factor for the development of clinical drug resistance. Knowledge of the amino acid motives, which are more prone to mutation in nature, could help in the timely recognition of emerging drug resistance during new drug therapy administration. Clinical data in drug-exposed populations and phenotypic resistance testing could confirm these results, as well as the analysis of a broader cohort of subjects, including also chronic HIV infections and further HIV-1 subtypes.

## Figures and Tables

**Table 1:** List of investigated amino acid variants possibly associated with natural resistance to Fostemsavir and Lenacapavir. FTR: Fostemsavir; LEN: Lenacapavir.

Drug	Gene	Mutation	Type of Resistance-Associated Variants	References
FTR	*env*	L116P/Q	in vitro selected	[6,7]
FTR	*env*	A204D	in vitro selected	[7]
FTR	*env*	S375H/I/M/N/T	clinically relevant resistance	[3,6,7,8]
FTR	*env*	S375Y	in vitro selected	[7]
FTR	*env*	M426L	clinically relevant resistance	[3,6,7,8]
FTR	*env*	M434I	clinically relevant resistance	[3,6,7,8]
FTR	*env*	M434K	in vitro selected	[7]
FTR	*env*	M475I	clinically relevant resistance	[3,6,7,8]
FTR	*env*	V506M	in vitro selected	[7]
LEN	*gag* (p24)	L56I	in vitro selected	[4,10]
LEN	*gag* (p24)	M66I	in vitro selected	[4,5,10]
LEN	*gag* (p24)	Q67H	in vitro selected	[4,5,10]
LEN	*gag* (p24)	K70N	in vitro selected	[4,10]
LEN	*gag* (p24)	N74D/S	in vitro selected	[4,5,10]
LEN	*gag* (p24)	A105T	in vitro selected	[4,10]
LEN	*gag* (p24)	T107N	in vitro selected	[4,10]

## Data Availability

The original data presented in the study are openly available in NCBI Sequence Reads Archive (SRA), in BAM file format, accession number PRJNA1144728.
